# Sodium-Glucose Cotransporter-2 Inhibitors in Type 2 Diabetes: From Metabolic Mechanisms to International Guidelines

**DOI:** 10.3390/antiox15050553

**Published:** 2026-04-27

**Authors:** Tamás Várkonyi, Krisztina Kupai, Hsu Lin Kang, Danica Matusovits-Varga, Dániel Priksz, Ákos Várkonyi, Csaba Lengyel, Anikó Pósa

**Affiliations:** 1 Department of Internal Medicine, Albert Szent-Györgyi Medical School, University of Szeged, 6703 Szeged, Hungary; varkonyi.tamas@med.u-szeged.hu (T.V.); kupai.krisztina@med.u-szeged.hu (K.K.);; 2 Department of Oral Biology and Experimental Dental Research, Faculty of Dentistry, University of Szeged, 6703 Szeged, Hungary; 3 Department of Pharmacology and Pharmacotherapy, Faculty of Medicine, Univerity of Debrecen, 4006 Debrecen, Hungary

**Keywords:** SGLT2, T2DM

## Abstract

Type 2 diabetes mellitus (T2DM) is a progressive metabolic disease that necessitates individualized therapeutic strategies focusing on both glycemic control and the mitigation of comorbidities. In recent years, sodium-glucose cotransporter-2 (SGLT2) inhibitors have emerged as a cornerstone of modern treatment due to their unique renal mechanism of action. This review summarizes the metabolic mechanisms, pleiotropic effects, and clinical significance of SGLT2 inhibitors in the management of T2DM. This review provides an updated overview of the metabolic and systemic effects of SGLT2 inhibition. By promoting glycosuria, SGLT2 inhibitors induce a negative energy balance that contributes to modest weight loss, improved body composition, and beneficial alterations in lipid metabolism. Beyond their metabolic effects, accumulating preclinical evidence suggests that SGLT2 inhibitors exert anti-inflammatory and antifibrotic actions, partly through modulation of macrophage polarization and attenuation of oxidative stress. The clinical utility of SGLT2 inhibitors is highlighted through the review of major cardiovascular and renal outcome trials, which confirm significant benefits in reducing heart failure hospitalizations and slowing the progression of chronic kidney disease. Finally, we integrate these findings into the context of the latest international guidelines while addressing safety profiles, the rationale for combination therapies, and the transition toward a personalized, risk-based management approach in T2DM.

## 1. Introduction

Type 2 diabetes mellitus (T2DM) is a chronic, progressive metabolic disease characterized by persistent hyperglycemia, leading to significant metabolic abnormalities and microvascular complications in the long term. The prevalence of the disease is steadily increasing worldwide, placing a significant burden on both individual health and healthcare systems. A significant proportion of patients with T2DM are overweight or obese, particularly with a predominance of visceral abdominal fat accumulation, which is closely associated with the degree of insulin resistance and the severity of metabolic dysregulation. Lifestyle factors such as a sedentary lifestyle, dyslipidemia, and arterial hypertension play a decisive role in the development and progression of the disease, but the importance of genetic predisposition cannot be ignored. Family history and belonging to certain high-risk ethnic groups clearly increase the likelihood of developing T2DM [1].

The complex pathophysiological background of the disease justifies the use of multi-targeted, individualized, metabolism-focused therapeutic strategies. Currently used antidiabetic drugs contribute to improving glycemic and metabolic control through different mechanisms of action by influencing the function of various target organs. Within this therapeutic spectrum, sodium-glucose cotransporter-2 (SGLT2) inhibitors have gained particular importance [2]. SGLT2 inhibitors, combined with dietary and lifestyle interventions, are an effective treatment option when used as monotherapy or in combination with other antidiabetic drugs.

Based on clinical trials, these drugs reduce glycated hemoglobin (HbA1c) levels to a clinically relevant extent without significantly increasing the risk of hypoglycemia. In addition, they have beneficial metabolic effects on weight loss, visceral fat reduction, hyperuricemia improvement, and favorable lipid metabolism [2,3]. Accordingly, the aim of this summary is to present the metabolic effects and clinical applications of SGLT2 inhibitors and their role in the latest diabetes guidelines, with a particular focus on their importance in the metabolic control of T2DM.

## 2. Metabolic Mechanisms of Action and Pleiotropic Metabolic Effects of SGLT2 Inhibitors

Renal D-glucose reabsorption along the proximal tubule is mediated by two Na^+^/glucose cotransporters. In the S1–S2 segments, SGLT2 accounts for ~90% of reabsorption and exhibits low affinity (K_0.5_ ≈ 6 mM) with a 1:1 Na^+^ coupling ratio. The remaining glucose is reabsorbed in the S3 segment by the high-affinity transporter SGLT1 (K_0.5_ ≈ 0.35 mM), which operates with a 2:1 Na^+^ coupling ratio [4]. By inhibiting SGLT2, SGLT2 inhibitors reduce the renal reabsorption of filtered glucose and lower the renal threshold for glucose, thereby increasing glucose excretion in the urine. As a result, HbA1c levels are reduced by an average of 0.6–1.0% [3]. Simultaneous inhibition of SGLT2-dependent glucose and sodium reabsorption increases sodium delivery to the distal tubules, which may lead to activation of tubuloglomerular feedback, reducing intraglomerular pressure. Although this process also has hemodynamic consequences, from a metabolic point of view, the reduction in the energy and oxygen requirements of tubular cells is particularly significant, which may contribute to a reduction in renal metabolic stress and glucotoxicity.

Under the influence of SGLT2 inhibitors, energy metabolism shifts partially towards gluconeogenesis and fatty acid oxidation, as well as ketone body-formation. As a result of glycosuria, approximately 60–90 g of glucose is excreted in the urine daily, resulting in an energy loss of about 200–300 kcal/day. This negative energy balance contributes to weight loss, but the actual weight loss observed in clinical trials (2–3 kg on average) falls short of the theoretically expected amount. This may be due to compensatory mechanisms, including increased energy intake and increased endogenous glucose production, which is likely mediated by glucagon released from α-cells [5]. Based on the analysis of body composition changes, in the short term, weight loss can be explained primarily by a decrease in body water content and, to some extent, lean body mass, while in longer-term treatments, a significant decrease in fat tissue can also be observed, which correlates with the duration of treatment. Reductions in muscle mass closely paralleled losses in body weight and fat mass. This concomitant decline underscores a significant risk for sarcopenia, requiring careful monitoring in patients already predisposed to physical frailty, especially in older individuals [6,7]. The use of SGLT2 inhibitors can also reduce visceral fat and liver fat [8,9]. Increased lipolysis and increased flux of free fatty acids may promote ketone-body formation, which may contribute to a reduction in liver fat and a favorable effect on non-alcoholic fatty liver disease (NAFLD).

Clinical data show that in patients with NAFLD, SGLT2 inhibitors can reduce liver enzyme levels (ALT, AST, GGT) [10], and some studies have also indicated a reduction in liver fibrosis [11]. Data on inflammatory markers are heterogeneous. In some studies, SGLT2 inhibitors reduced CRP levels, while changes in other inflammatory cytokines (TNF-α, IL-1β, IL-6, IL-10) were not significant. However, meta-analyses suggest that adiponectin levels may increase, while IL-6 and TNFR1 (tumor necrosis factor receptor 1) concentrations may decrease [12]. These results suggest that the anti-inflammatory effect of SGLT2 inhibitors is indirect and may be closely related to a reduction in adipose tissue.

The effect of SGLT2 inhibitors on lipid metabolism is complex. Based on meta-analyses, treatment increases total cholesterol, LDL, and HDL cholesterol levels while reducing triglyceride concentrations [13]. Experimental data suggest that these changes may be related to increased lipoprotein lipase activity and decreased ANGPTL4 (angiopoietin-like protein 4) expression, which promotes the conversion of VLDL to LDL and reduces postprandial lipemia [14,15,16,17]. Although a slight increase in LDL cholesterol can be observed, these lipid changes are accompanied by favorable changes in body composition and visceral fat accumulation. Overall, the mechanism of action of SGLT2 inhibitors goes beyond glycemic control: the negative energy balance induced by glycosuria, the rearrangement of substrate utilization, the reduction in adipose tissue and liver fat content, and the favorable changes in lipid and inflammatory parameters all contribute to the improvement of the complex metabolic dysregulation of T2DM.

## 3. The Mechanisms of Action of SGLT2 Inhibitors in Experimental Models Involve Complex, Closely Intertwined Metabolic, Inflammatory, and Tissue-Protective Processes

Preclinical studies have clearly demonstrated that SGLT2 inhibitor treatment promotes the polarization of M2-type, alternatively activated macrophages in white adipose tissue, thereby reducing chronic inflammation and insulin resistance associated with obesity in obese mice. Dapagliflozin and canagliflozin had a beneficial effect on metabolic parameters measured in NAFLD models, reducing liver fat accumulation, inflammation, and fibrosis in both leptin receptor-deficient db/db mice and other NAFLD models [18,19]. Treatment reduced the proportion of classically activated M1 macrophages, while increasing the presence of M2 macrophages in liver tissue, which was associated with a reduction in the inflammatory response and structural and functional protection of hepatocytes [20]. These results suggest that SGLT2 inhibitors, in addition to their glucose-lowering effect, are also capable of directly modulating the macrophage population in the liver. Empagliflozin treatment has been shown to increase fatty acid utilization, reduce inflammatory activity, and improve insulin sensitivity in obese mice [18,20].

The cardiovascular effects of SGLT2 inhibitors have also been extensively studied in preclinical models. Dapagliflozin treatment in diabetic heart models reduced left ventricular remodeling, improved both diastolic and systolic function, and significantly reduced interstitial collagen accumulation. Based on in vitro studies, the cardioprotective effect of dapagliflozin is independent of blood glucose reduction and inhibits the proliferation, activation, and collagen synthesis of cardiac fibroblasts in a dose-dependent manner [20,21]. The antifibrotic effect is mediated by a signaling mechanism involving AMP-activated protein kinase alpha subunit (AMPKα). The latest experimental results have also confirmed significant antioxidant activity in cardiac muscle cells in the case of SGLT2 inhibitors [22]. Dapagliflozin significantly inhibited the increased expression of NADPH oxidase 4 (NOX4) in diabetic rats, reducing oxidative stress, fibrosis, and pathological cardiac remodeling. Byrne et al. studied the effect of empagliflozin on induced heart failure in male C57BL/6 non-diabetic mice. Fourteen days of empagliflozin treatment improved systolic function, while progressive deterioration of cardiac function was observed in the control group [23]. Li et al. demonstrated in KK-Ay mice with type 2 diabetes mellitus (T2DM) that empagliflozin reduces myocardial fibrosis and oxidative stress through inhibition of transforming growth factor beta (TGF-β) and the Smad signaling pathway [24]. Penning et al. demonstrated in a mouse model induced by severe hypercholesterolemia and atherosclerosis that empagliflozin reduces atherosclerotic plaque size, lipid levels, and macrophage accumulation [25]. Lee et al. demonstrated in spontaneously hypertensive rats that empagliflozin improves atrial and ventricular remodeling and has a pronounced renoprotective effect without significantly affecting plasma glucose levels. Lee et al. further analyzed the vascular and systemic effects and confirmed that dapagliflozin reduces arterial stiffness, improves endothelial and smooth muscle function, and favorably modifies the composition of the gut microbiota in T2DM mice [26,27].

With regard to skeletal muscle metabolism, Nambu et al. demonstrated that empagliflozin improves endurance and enhances mitochondrial fatty acid oxidation in heart-failure mice, which was associated with an increase in beta-hydroxybutyrate (β-OHB) levels [28]. Oshima et al. observed similar results in Otsuka Long Evans Tokushima Fatty (OLETF) rats with myocardial infarction, where an increase in β-OHB levels was accompanied by preservation of adenosine triphosphate (ATP) levels [29].

Baker et al. demonstrated in normoglycemic pigs that canagliflozin preserves cardiac contractile function during acute regional myocardial infarction [30]. Zhang et al. and Santos Gallego et al. demonstrated in non-diabetic pig models that dapagliflozin and empagliflozin improve cardiac function, reduce neurohormonal activation, and induce favorable metabolic changes [31]. Lee et al. demonstrated in normoglycemic, atherosclerotic animals that dapagliflozin reduces the development of atherosclerotic lesions, intimal proliferation, and the expression of proinflammatory markers. In a model of hypertrophic cardiomyopathy, in mice carrying the myosin R403Q mutation, sixteen weeks of empagliflozin treatment improved cardiac energetics, normalized intracellular sodium concentration, improved mitochondrial function, reduced left ventricular hypertrophy and fibrosis, and improved diastolic function and contractile reserve [27,32]. Empagliflozin improved the metabolic link between glycolysis and glucose oxidation and enhanced mitochondrial ATP synthesis, consistent with findings reported in other diabetic and non-diabetic cardiomyopathy models [29,33,34].

## 4. The Therapeutic Spectrum of SGLT2 Inhibitors in Light of Clinical Outcome Studies

Phase III large clinical outcome trials have consistently demonstrated that SGLT2 inhibitors also have moderate but clinically relevant metabolic effects, particularly in terms of glycemic control, body weight, and, to some extent, lipid profile. In the EMPA-REG OUTCOME trial, after the 12-week protocol-specified stable glycemic-treatment phase, empagliflozin reduced HbA1c by **0.54%** (10 mg) and **0.60%** (25 mg) relative to placebo. During extended follow-up, the HbA1c differences were **−0.42%** and **−0.47%** at 94 weeks, and **−0.24%** and **−0.36%** at 206 weeks. The treatment was also associated with modest reductions in body weight and small increases in both LDL-cholesterol and HDL-cholesterol. [35].

The integrated analysis of the CANVAS Program (canagliflozin) also provides a good numerical overview of the “metabolic package”: the average difference in HbA1c was 0.58 percentage points; the difference in body weight was 1.60 kg, and HDL and LDL cholesterol increased; meanwhile, their ratio remained unchanged [36]. In the DECLARE TIMI 58 study of dapagliflozin, the difference in HbA1c was 0.42 percentage points and the difference in weight loss was 1.8 kg, indicating stable glycemic and weight effects even in a broader population with only risk factors [37].

In the VERTIS CV study of ertugliflozin, the change in HbA1c at week 18 was a decrease of 0.70 and 0.72 percentage points (5 mg and 15 mg, respectively) compared to baseline, while with placebo it was 0.22 percentage points, and at 1 year, body weight decreased by an average of 2.4 kg in the 5 mg group [38]. In the CREDENCE study, the use of canagliflozin was associated with a sustained reduction in HbA1c and moderate weight loss, while changes in lipid parameters remained modest and did not worsen the overall cardiometabolic risk profile [39].

In the DAPA CKD study, dapagliflozin showed a significant HbA1c and weight reduction effect in the subgroup with diabetes mellitus, which also proved to be metabolically neutral in the non-diabetic population [40]. Finally, the EMPA KIDNEY study with empagliflozin confirmed that glycemic control remains stable during treatment, body weight is reduced, and only minimal, clinically insignificant shifts in the lipid profile occur, further confirming the favorable metabolic safety of SGLT2 inhibitors [41]. Canagliflozin is one of the first approved representatives of the SGLT2 inhibitor group, which can be used as a supplement to diet and exercise to improve glycemic control in adults and children over 10 years of age with T2DM. It has been shown to reduce major adverse cardiovascular events (MACE) in adults with T2DM who have established cardiovascular disease. In addition, it reduces the risk of end-stage renal disease (ESKD) and hospitalization due to heart failure in patients with T2DM and diabetic nephropathy characterized by albuminuria exceeding 300 mg per day. These indications are supported by large-scale cardiovascular and renal outcome studies. The CANVAS Program (CANVAS and CANVAS-R trials) demonstrated that canagliflozin significantly reduced the relative risk of the composite endpoint of MACE by 14% in patients with T2DM and high cardiovascular risk [35]. The CREDENCE trial provided decisive evidence of renoprotective effects [36].

Dapagliflozin has been shown to reduce the risk of cardiovascular death, hospitalization for heart failure, and acute heart failure care in patients with reduced or preserved ejection fraction heart failure, regardless of diabetes status. In chronic kidney disease, it reduces the risk of sustained decline in eGFR, development of ESKD, and cardiovascular mortality and hospitalization for heart failure. The DECLARE–TIMI 58 trial, involving more than 17,000 patients with T2DM, showed that although dapagliflozin did not significantly reduce the incidence of MACE, it significantly reduced the risk of cardiovascular mortality [37]. The DAPA-HF and DELIVER studies confirmed the efficacy of dapagliflozin in the spectrum of heart failure with preserved ejection fraction, while a meta-analysis also supported a significant reduction in cardiovascular and all-cause mortality. The DAPA-CKD study demonstrated a clear renoprotective effect, regardless of the presence of diabetes [38].

Empagliflozin can be used in adults and children over 10 years of age with T2DM to improve glycemic control and is also approved for reducing the risk of cardiovascular mortality and hospitalization due to heart failure. The EMPEROR-Reduced and EMPEROR-Preserved studies demonstrated its efficacy across the entire spectrum of heart failure, while the EMPULSE study also supported its early use in acute decompensated heart failure [39].

Ertugliflozin is currently only approved for improving glycemic control in adult patients with T2DM. The VERTIS-CV trial confirmed its cardiovascular safety, but did not show a significant reduction in risk in terms of MACE or hospitalization for heart failure [40]. Bexagliflozin is also only approved for the treatment of glycemic indications in adult patients with T2DM. Its cardiovascular safety has been confirmed in a dedicated outcome trial, but it does not have any cardiovascular or renal risk reduction indications [41].

As a dual SGLT1/SGLT2 inhibitor, sotagliflozin reduces the risk of cardiovascular death, hospitalization for heart failure, and acute heart failure care in heart failure, including forms with reduced and preserved ejection fraction. Its efficacy has been demonstrated in the SOLOIST-WHF and SCORED studies in patients with T2DM and chronic kidney disease.

## 5. Safety Profile and Clinically Relevant Adverse Events of SGLT2 Inhibitors

The side effect profile associated with the use of SGLT2 inhibitors is well defined and closely related to the specific, primarily renal mechanism of action of this class of drugs. The most commonly observed adverse events include transient deterioration of renal function, which manifests as an increase in serum creatinine levels and an initial decrease in estimated eGFR after initiation of treatment. These changes are typically hemodynamic in origin, explained by a decrease in glomerular intravascular pressure, and in many cases stabilize or are reversible with continued therapy [42,43,44].

Due to the mechanism of action of SGLT2 inhibitors, which is associated with increased glucose excretion, there is an increased risk of urinary tract infections and genital mycotic infections. The latter occur more frequently in female patients, in cases of poor glycemic control, and in patients with a history of similar infections. Based on retrospective studies and meta-analyses, SGLT2 inhibitors pose a greater risk of genital infections compared to other antidiabetic drugs [45]. The rate of genital fungal infections was particularly higher with canagliflozin, and in some cases, treatment had to be discontinued. Empagliflozin was also associated with an increased risk of genital and urinary tract infections compared to placebo, metformin, or sitagliptin. Due to increased osmotic diuresis, SGLT2 inhibitors may reduce intravascular volume, which in sensitive patient groups—particularly the elderly, those taking diuretics, or those with low blood pressure—may lead to orthostatic hypotension and dehydration. These effects can usually be prevented with adequate fluid intake, dose adjustment, and patient selection.

Some clinical studies have also indicated an increased risk of bone fractures, primarily in association with the use of canagliflozin. The FDA has confirmed the warning about the increased risk of fractures with canagliflozin and has released new information about bone density loss [46]. In the CANVAS program, at least one fracture event was recorded in 496 subjects with T2DM and cardiovascular disease. In contrast, in the CREDENCE study, the fracture rate observed with canagliflozin did not differ significantly from that in the placebo group [46]. Based on several meta-analyses, SGLT2 inhibitors did not increase the risk of fractures overall compared to placebo, and canagliflozin did not show an increased risk of fractures compared to GLP-1 receptor agonists [47].

A rare but clinically significant side effect is the risk of euglycemic diabetic ketoacidosis (euDKA), which can occur during SGLT2 inhibitor treatment even with normal or only moderately elevated blood glucose levels [48]. Shortly after the FDA issued its warning, the manufacturer of canagliflozin announced the total incidence of DKA in its clinical trial database [49]. A significant proportion of the cases involved T1DM, an indication for which SGLT2 inhibitors are not approved, or a diagnosis of latent autoimmune diabetes mellitus in adults. Peters et al. identified additional risk factors for the development of euDKA, including reduction or discontinuation of insulin, major surgery, alcohol consumption, and low-carbohydrate or ketogenic diets [48]. Based on the available data, the overall risk-benefit ratio justifies the continued use of SGLT2 inhibitors, but professional statements recommend suspending treatment at least 24–72 h before planned major surgery and that sudden reductions in insulin doses, excessive alcohol consumption, and ketogenic diets should be avoided [50].

Clinical trials have also shown that the safety profiles of individual SGLT2 inhibitors may differ. Canagliflozin (100 and 300 mg) increased the incidence of ketoacidosis-related events during an 18-week treatment period [51]. The use of dapagliflozin (5 and 10 mg) was associated primarily with mild side effects, such as nasopharyngitis and urinary tract infections, while the incidence of severe hypoglycemia was lower, but the risk of DKA still called for caution [52,53]. During 52 weeks of treatment with sotagliflozin (400 mg), an increased risk of both DKA and hypoglycemia was observed [54,55]. In contrast, the risk of DKA with empagliflozin was dose-dependent (10 mg: 4.3%; 25 mg: 3.3%; 2.5 mg: 0.8%), while not associated with an increased risk of hypoglycemia [56]. No significant safety concerns were observed during 24 weeks of treatment with ipragliflozin (50 mg), suggesting a favorable tolerability profile [57].

## 6. Possibilities for Combination Therapy and Fixed-Dose Combinations in the Use of SGLT2 Inhibitors

The use of SGLT2 inhibitors in combination therapy is of considerable clinical interest in the treatment of both T1DM and T2DM, as they may have beneficial effects on body weight, insulin requirements, and certain cardiometabolic risk factors in addition to improving glycemic control. When used as an adjunct to insulin therapy in T1DM, SGLT2 inhibitors may reduce blood glucose levels, body weight, and exogenous insulin requirements, but their use should be considered with caution due to potential side effects, particularly an increased risk of DKA [58,59].

A small study suggested that the combination of dapagliflozin and a glucagon receptor antagonist may enhance the glucose-lowering effect and reduce the risk of DKA, but these results need to be further confirmed due to the limited number of patients [60]. Given that SGLT2 inhibitors have a significant antihyperglycemic effect on their own, their combination with other antidiabetic agents is of particular clinical importance. It can be assumed that there are additive interactions between the mechanisms of action of the individual drug groups, but further studies are needed to determine the optimal use of these combinations.

Combination with GLP-1 receptor agonists does not increase the risk of hypoglycemia, and the favorable cardiovascular and renal effects are maintained due to the independent mechanisms of action of the two drug classes [61,62]. When combined with pioglitazone, SGLT2 inhibitors show additive benefits in terms of weight loss and reduced risk of hypoglycemia, and may also reduce the risk of heart failure [63]. The combination with metformin is particularly well established in the treatment of T2DM, as the two agents exert their effects through complementary pathophysiological mechanisms. This combination may have anti-inflammatory effects, does not increase the risk of fractures, and does not adversely affect the cardiovascular benefits of SGLT2 inhibitors [64]. Triple therapy (SGLT2 inhibitor, metformin, and DPP-4 inhibitor) may provide more effective glycemic control, but may be associated with an increased risk of genital infections [65].

To improve therapeutic adherence, several fixed-dose combination (FDC) products have been marketed since 2014, starting with canagliflozin-metformin, followed by dapagliflozin-metformin and empagliflozin-metformin in various immediate- and extended-release formulations. FDCs are available in a range of strengths, allowing for individualized dosing, gradual titration, and adjustments based on renal function. The clinical efficacy of FDCs has been confirmed in several Phase III studies: the combination of canagliflozin and metformin resulted in an approximately 1.8% reduction in HbA1c over 26 weeks [66], while the initial combinations of empagliflozin-metformin and dapagliflozin-metformin resulted in similar HbA1c reductions of approximately 2% [42,43]. Overall, the combination use of SGLT2 inhibitors—including FDCs with insulin, other oral antidiabetic drugs, and metformin—is an effective and rational therapeutic strategy for both T1DM and T2DM. However, careful consideration of the balance between efficacy and safety, as well as individualization of treatment decisions, is essential when using them.

## 7. The Role of SGLT2 Inhibitors in International Guidelines

According to the American Diabetes Association (ADA) treatment guidelines, the recommended HbA1c target for most adult patients with T2DM is below 7.0%, which can be individualized taking into account individual risk factors, comorbidities, and the risk of hypoglycemia [1]. The American Association of Clinical Endocrinologists and the American College of Endocrinology (AACE/ACE) set a stricter target and recommend an HbA1c value below 6.5% for patients for whom this is safely achievable.

However, the AACE/ACE emphasizes that although more intensive glycemic control reduces the risk of microvascular complications, in certain patient groups it may also be associated with an increase in adverse events, particularly hypoglycemia. HbA1c targets can be achieved through a combination of lifestyle interventions and blood glucose-lowering medications with different mechanisms of action.

The ADA guidelines initially recommend monotherapy for patients with HbA1c < 9.0% at the start of treatment, while the AACE/ACE only recommends monotherapy if the baseline HbA1c is <7.5%. Both organizations consider metformin to be the first-line treatment, but other agents, including SGLT2 inhibitors, may be used as alternatives in cases of intolerance or contraindications. According to the ADA recommendation, if the HbA1c target value is not achieved after three months of metformin monotherapy, dual therapy is indicated with the addition of another antihyperglycemic agent. SGLT2 inhibitors are one of the six main drug classes recommended by the ADA, alongside thiazolidinediones, sulfonylureas, DPP-4 inhibitors, GLP-1 receptor agonists, and basal insulin. In cases where the baseline HbA1c is ≥9.0%, dual therapy can be initiated without first trying metformin [1].

The Standards of Care in Diabetes-2026 [67] guidelines published in Diabetes Care maintain the above glycemic targets and stepwise therapeutic logic, but shift the focus of therapy selection even more emphatically toward comorbidities and risk profiles. According to the ADA 2026 approach [67], the treatment of T2DM is a complex condition requiring continuous care, in which risk reduction strategies go beyond glycemic management, and drug selection must consistently reflect the patient’s individual organ risk and risk of progression. Accordingly, the role of SGLT2 inhibitors in the ADA 2026 standards is not limited to HbA1c reduction, but is also systematically integrated into situations where the key considerations are slowing the progression of chronic kidney disease and providing targeted protection for high-risk patient groups.

## 8. Role in International Cardiology and Nephrology Guidelines

Based on their high efficacy and favorable clinical outcome data, SGLT inhibitors have received strong recommendations in international guidelines for major cardiovascular, renal, and diabetic conditions. The 2024 Kidney Disease: Improving Global Outcomes (KDIGO) guidelines strongly recommend initiating SGLT2 inhibitor therapy in patients with T2DM and chronic kidney disease with an estimated eGFR of at least 20 mL/min/1.73 m^2^. Continuation of treatment is recommended even if the eGFR falls below 20 mL/min/1.73 m^2^, except in cases of intolerance or initiation of renal replacement therapy [68].

The ADA treatment standards emphasize the use of cardioprotective and renoprotective therapies in T2DM, regardless of glycemic targets. In cases of established atherosclerotic cardiovascular disease or high risk thereof, the ADA recommends the use of agents that have been shown to reduce major cardiovascular events, such as GLP-1 receptor agonists and/or SGLT2 inhibitors. In the presence of heart failure, regardless of ejection fraction, the use of SGLT2 inhibitors is recommended to reduce mortality and hospitalization.

The guidelines of the European Society of Cardiology (ESC) clearly support the use of SGLT2 inhibitors in patients with T2DM and heart failure. In heart failure with reduced ejection fraction, SGLT2 inhibitors are recommended for all patients to reduce hospitalization for heart failure and cardiovascular mortality [69]. Based on the 2023 updates, this recommendation also extends to heart failure with mildly reduced and preserved ejection fraction [70]. The 2024 ESC guidelines on chronic coronary syndromes also recommend the use of SGLT2 inhibitors in T2DM, regardless of HbA1c levels and other glucose-lowering therapies [71].

According to the recommendations of the American College of Cardiology (ACC), SGLT2 inhibitors are recommended for patients with T2DM and chronic coronary artery disease to reduce the risk of MACE. In heart failure with reduced ejection fraction, the use of SGLT2 inhibitors is strongly recommended, regardless of the presence of diabetes, as they reduce mortality and hospitalization and improve quality of life. A beneficial effect can also be observed in heart failure with preserved ejection fraction, although their cost-effectiveness in this group is currently less clear [72,73].

In line with the above international framework, the ADA 2026 Standards of Care specifically highlight risk-based management of chronic kidney disease (CKD) progression and recommend SGLT2 inhibitors for individuals at high risk of CKD progression (e.g., in the presence of albuminuria or documented eGFR decline).

## 9. Conclusions

SGLT2 inhibitors extend beyond traditional antihyperglycemic therapy by providing a metabolism-based approach to T2DM management. In addition to improving glycemic control, they confer clinically relevant cardiovascular and renal protection, largely independent of their glucose-lowering effects. Accordingly, current international guidelines position this drug class centrally, particularly in patients with heart failure or chronic kidney disease.

Emerging evidence suggests that their benefits may also involve modulation of cellular energetics, mitochondrial function, and inter-organ metabolic crosstalk, highlighting mechanisms that extend beyond classical glucotoxicity reduction. In parallel, novel therapeutic strategies are being explored, including combination approaches with incretin-based therapies and dual SGLT1/2 inhibition, which may further enhance metabolic and cardiorenal outcomes.

Future priorities include further individualization of therapy, identification of predictive metabolic biomarkers, and clarification of long-term safety and organ-protective mechanisms. The integration of digital health tools and real-time metabolic monitoring may also refine patient selection and treatment optimization. Given the risk of euglycemic diabetic ketoacidosis, enhanced monitoring strategies may be warranted; emerging technologies such as continuous ketone monitoring systems could enable earlier detection of ketosis, although clinical validation remains limited.

**Figure 1:**
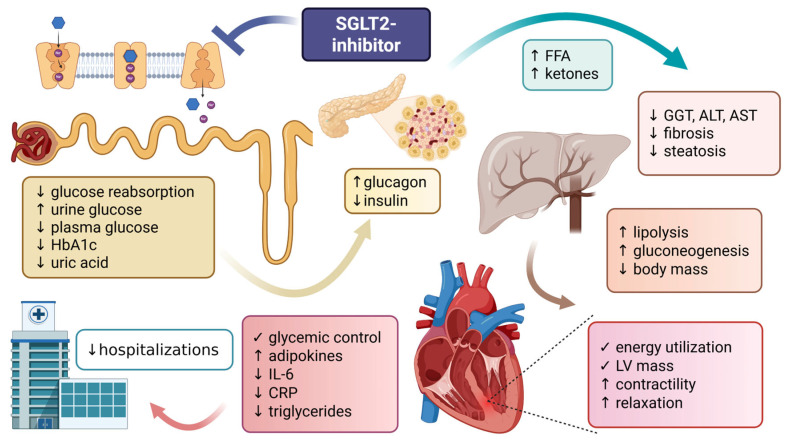
Metabolic effects of SGLT2 inhibitors. (Created in BioRender. Krisztina, D. (2026) https://BioRender.com/sehu28u).

Overall, SGLT2 inhibitors represent a key component of personalized, metabolism-oriented diabetes care and continue to evolve as a platform for mechanism-based therapeutic innovation (Figure 1).

## Data Availability

No new data were created or analyzed in this study. Data sharing is not applicable to this article.
